# Evaluation of circulating levels of CCN2/connective tissue growth factor in patients with ST-elevation myocardial infarction

**DOI:** 10.1038/s41598-017-12372-w

**Published:** 2017-09-20

**Authors:** Vibeke Ritschel, Christian Shetelig, Ingebjørg Seljeflot, Shanmuganathan Limalanathan, Pavel Hoffmann, Sigrun Halvorsen, Harald Arnesen, Jan Eritsland, Geir Øystein Andersen

**Affiliations:** 1 Department of Cardiology, Oslo University Hospital Ullevål, Oslo, Norway; 2 Center for Clinical Heart Research, Oslo University Hospital Ullevål, Oslo, Norway; 3 Center for Heart Failure Research, Oslo, Norway; 4 Faculty of Medicine, University of Oslo, Oslo, Norway; 5 LHL Clinics, Feiring Heart Clinic, Feiring, Norway; 6 Section of Interventional Cardiology, Oslo University Hospital Ullevål, Oslo, Norway

## Abstract

CCN2/Connective tissue growth factor seems to be involved in development of cardiac hypertrophy and fibrosis, but a possible cardioprotective role in left ventricular (LV) remodelling following myocardial infarction has also been suggested. The main objectives of the study were therefore to investigate whether circulating CCN2 levels were associated with infarct size, LV function, adverse remodelling or clinical outcome in two cohorts of patients with ST-elevation myocardial infarction (STEMI). CCN2 was measured in 988 patients 18 hours after PCI and clinical events were recorded after 55 months in the BAMI cohort. In the POSTEMI trial, serial measurements of CCN2 were performed in 258 STEMI patients during index hospitalisation and cardiac magnetic resonance imaging was performed in the acute phase and after 4 months. Clinical events were also recorded. There were no significant associations between levels of CCN2 and infarct size, LV ejection fraction, changes in LV end-diastolic or end-systolic volume, myocardial salvage or microvascular obstruction. There were no significant associations between CCN2 levels and clinical events including mortality, in either of the study cohorts. In conclusion, circulating levels of CCN2 measured in the acute phase of STEMI were not associated with final infarct size, left ventricular function or new clinical events.

## Introduction

Adverse left ventricular (LV) remodelling, impaired myocardial salvage and heart failure development remain unresolved issues in patients with acute ST-elevation myocardial infarction (STEMI) despite modern reperfusion therapy. Although the underlying mechanisms are extensively studied, specific therapeutic approaches remain challenging.

CCN2/Connective tissue growth factor, which is upregulated in response to transforming growth factor beta 1 (TGF-β1)^1,2^, is a member of the CCN family of matricellular proteins^3^. CCN2 is involved in a variety of biological processes including extracellular matrix synthesis, cell proliferation and angiogenesis. It has been implicated in the pathogenesis of cardiac hypertrophy and fibrosis^4–7^, and has been found to be upregulated in models of cardiac pressure-overload induced heart failure^6,8^ and myocardial infarction (MI)^9,10^, suggesting a role in cardiac remodelling. Although it is mainly described as a pro-fibrotic marker, some experimental models involving cardiac-restricted overexpression of CCN2 and post-ischaemic administration of recombinant CCN2 have suggested that CCN2 may be cardioprotective by attenuating LV remodelling^11,12^, increasing tolerance to ischaemia-reperfusion (IR) injury^13^ and reducing infarct size^14^. Circulating levels of CCN2 have been shown to be associated with heart failure in clinical studies^15^, however, the role of endogenous CCN2 in acute MI in humans remains unclear. Patients with increase in serum levels of CCN2 after MI were reported to have improved LV function and possibly attenuated LV remodelling in a small human study^11^. There is however, a lack of information from clinical studies with adequate sample size on circulating levels of CCN2 during the acute phase of MI, the effect of reperfusion therapy on CCN2 levels and any possible associations between CCN2 and IR-injury, ischaemic postconditioning, adverse remodelling, as well as later clinical outcome.

We therefore wanted to investigate, whether levels of circulating CCN2 were associated with myocardial injury, remodelling, and clinical outcome in two separate cohorts of STEMI patients treated by primary percutaneous coronary intervention (PCI). Possible associations with myocardial IR-injury, including myocardial salvage and ischaemic postconditioning, adverse remodelling and final infarct size assessed by cardiac magnetic resonance imaging (CMR) were explored in the Postconditioning in ST-Elevation Myocardial Infarction (POSTEMI) study, while studying associations between circulating CCN2 levels and clinical outcome was the main objective of the Biobanking in Acute Myocardial Infarction (BAMI) study.

## Results

Data were obtained from 258 (POSTEMI) and 988 (BAMI) patients from the two study populations, respectively. Median values of CCN2 measured at Day 1 (median 18 hours after PCI in both populations) were 23.3 ng/ml in the POSTEMI cohort and 27.1 ng/ml in the BAMI cohort. Characteristics of the study populations according to high or low CCN2 levels at Day 1 (dichotomised at median) are shown in Table 1. In both populations, low levels of CCN2 were significantly associated with current smoking. Additionally, high levels of CCN2 were associated with treated hypertension and higher body mass index, but these findings were not consistent across the two populations. There was no significant difference in peak troponin T levels between patients with high or low CCN2 levels.

**Table 1:** Characteristics of the study populations according to CCN2 levels measured at Day 1 (above or below median value) in two cohorts of STEMI patients.

	POSTEMI (n = 249)			BAMI (n = 988)		
	CCN2 ≤ median (n = 125)	CCN2 > median (n = 124)	p-value	CCN2 ≤ median (n = 495)	CCN2 > median (n = 493)	p-value
Age (years)	60 (53, 66)	61 (54, 68)	0.53	60 (52,69)	61 (54,71)	0.08
Female gender	23 (18.4%)	20 (16.1%)	0.64	88 (17.8%)	109 (22.1%)	0.40
Body mass index (kg/m^2^)	26.0 (24.1, 28.6)	27.2 (24.9, 29.4)	**0.03**	26.4 (24.1, 29.3)	26.6 (24.3, 29.1)	0.46
Current smoker	80 (64.0%)	45 (36.3%)	**<0.001**	277 (56.0%)	183 (37.1%)	**<0.0001**
*Previous disorders:*						
Myocardial infarction	—	—	—	61 (12.3%)	59 (12.0%)	0.94
Hypertension	33 (26.4%)	36 (29.0%)	0.64	147 (29.7%)	196 (39.8%)	**0.01**
Hypercholesterolaemia	11 (8.8%)	12 (9.7%)	0.81	69 (13.9%)	71 (14.4%)	0.91
Diabetes mellitus	9 (7.2%)	6 (4.8%)	0.43	58 (11.7%)	72 (14.6%)	0.21
Heart failure	0 (0%)	0 (0%)	—	8 (1.62%)	12 (2.43%)	0.49
Cerebrovascular disease	5 (4.0%)	2 (1.6%)	0.26	25 (5.05%)	20 (4.06%)	0.55
*Biochemical analyses:*						
Peak troponin T (ng/L)	5933 (3292, 11555)	5882 (3332, 10453)	0.57	3840 (1760, 7080)	3840 (1685, 7205)	0.96
Peak CRP^a^ (mg/L)	20 (8, 55)	20 (7, 46)	0.94	14 (7, 32)	13 (8, 31)	0.95
Admission total cholesterol (mmol/L)	5.3 (4.7, 5.9)	5.1 (4.5, 6.0)	0.14	4.8 (4.0, 5.6)	4.8 (4.1, 5.5)	0.50
Admission glucose (mmol/L)	8.0 (6.8, 9.5)	7.9 (6.6, 9.3)	0.96	7.3 (6.3, 9.0)	7.6 (6.4, 9.1)	0.88
Admission HbA1C (%)	6.0 (5.7, 6.2)	6.0 (5.7, 6.2)	0.76	5.9 (5.7, 6.3)	5.9 (5.6, 6.3)	0.58
Admission NT-proBNP^b^ (ng/L)	8 (5, 21)	12 (5, 23)	0.13	28 (9, 122)	38 (12, 126)	**0.04**
Admission creatinine (μmol/L)	69 (61, 78)	70 (65, 83)	0.08	71 (61, 84)	73 (63, 86)	0.17
*Infarct characteristics:*						
Time from symptom to PCI^c^ (min)	163 (114, 242)	200 (133, 282)	0.01	240 (180, 420)	240 (180, 360)	0.25
Anterior MI^d^	56 (44.8%)	66 (53.2%)	0.18	198 (40.0%)	229 (46.5%)	0.05

CCN2 was measured median 18 hours after PCI. Continuous data are presented as median (25^th^, 75^th^ percentiles) and categorical data as numbers (%). ^a^CRP: C-reactive protein, ^b^NT-proBNP: N-terminal pro-B-type natriuretic peptide, ^c^PCI: Percutaneous coronary intervention, ^d^Infarct localisation – Anterior myocardial infarction (MI) vs inferior or posterior MI. P-values obtained by Mann-Whitney U test for continuous variables, Chi-square test for categorical variables, significant p-values (p < 0.05) highlighted in bold.

### Temporal profile of CCN2 during STEMI and association with ischaemic postconditioning

**Figure 1:**
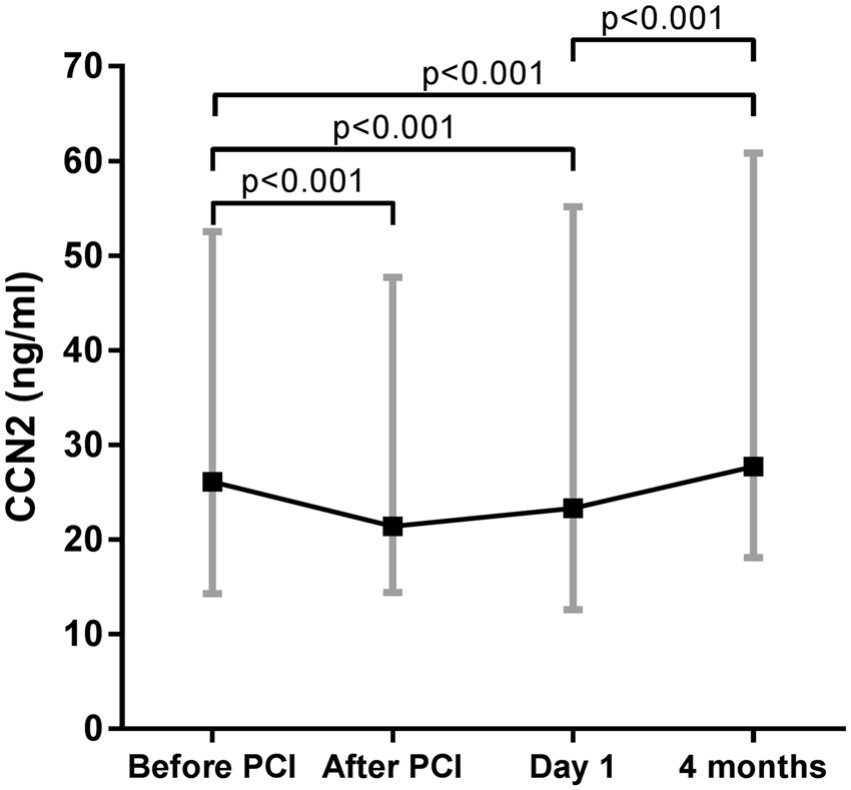
Temporal profile of CCN2 during STEMI in the POSTEMI cohort. CCN2 was measured in 258 patients with STEMI from blood sampled before and immediately after the PCI procedure, at Day 1 (median 18.3 hours after PCI), and at 4-month follow-up. Data are presented as median (boxes) with 25^th^ and 75^th^ percentile (whiskers). P-values obtained from Wilcoxon signed rank test.

There was a significant decline in CCN2 levels during PCI, while there was a significant increase from after PCI to Day 1, and a further increase to 4-month follow-up (Fig. 1). Patients treated with ischaemic postconditioning (iPost) and conventional PCI had similar CCN2 levels (before PCI, median values 26.1 vs 26.6 ng/ml (p = 0.97); after PCI, 20.3 vs 22.6 ng/ml (p = 0.68); Day 1, 20.8 vs 25.7 ng/ml (p = 0.77); 4 months 27.4 vs 29.9 ng/ml (p = 0.86)). Consequently, the POSTEMI population was analysed as a whole.

### Associations between CCN2 levels and myocardial injury and function assessed by CMR

We did not find any significant associations between circulating levels of CCN2 at any sampling point and infarct size, LV ejection fraction (LVEF), change in indexed LV end-diastolic volume (LVEDVi) and LV end-systolic volume (LVESVi), myocardial salvage or microvascular obstruction (MVO), respectively, neither in correlation analyses with CCN2 analysed as a continuous variable (Supplementary Table S1), nor in group analyses with CCN2 dichotomised at median value (Table 2).

**Table 2:** Infarct size, microvascular obstruction and left ventricular function and volumes assessed by CMR in the acute phase and after 4 months according to CCN2 values (above or below median) in 249 STEMI patients (POSTEMI cohort).

	CCN2 - Before PCI			CCN2 - After PCI			CCN2 - Day 1			CCN2–4 months		
	≤median	>median	p	≤median	>median	p	≤median	>median	p	≤median	>median	p
**CMR** ^**a**^ **in the acute phase**												
Infarct size (% of LV^b^ mass)	16.9 (10.7, 28.8)	18.0 (11.3, 28.5)	0.96	16.4 (10.9, 28.1)	18.0 (10.9, 28.5)	0.92	16.9 (10.7, 28.4)	18.4 (11.6, 29.0)	0.77			
Ejection fraction (%)	51 (42, 59)	51 (44, 58)	0.94	51 (42, 59)	51 (44, 57)	0.75	51 (42, 59)	51 (44, 56)	0.64			
Area at risk (% of LV)	43.3 (33.9, 53.7)	41.5 (33.4, 53.5)	0.57	43.5 (33.9, 54.9)	40.7 (33.4, 52.1)	0.32	43.6 (34.6, 53.3)	41.4 (32.8, 54.0)	0.35			
Presence of MVO^c^	54 (48.2%)	59 (53.2%)	0.46	51 (45.5%)	60 (54.1%)	0.20	50 (45.9%)	58 (54.2%)	0.22			
**CMR after 4 months**												
Infarct size (% of LV mass)	14.3 (6.9, 22.1)	13.5 (7.9, 23.3)	0.89	14.3 (7.7, 22.3)	13.5 (7.7, 23.0)	0.70	14.2 (6.8, 22.7)	13.6 (8.0, 21.9)	0.76	14.3 (8.3, 23.1)	13.7 (7.6, 22.4)	0.51
Ejection fraction (%)	57 (50, 62)	54 (47, 63)	0.49	57 (49, 63)	55 (49, 63)	0.63	57 (49, 62)	55 (48, 63)	0.71	56 (49, 62)	55 (48, 63)	0.98
Myocardial salvage (%)	54.8 (39.9, 70.6)	48.4 (35.4, 62.7)	0.11	54.8 (39.9, 69.6)	48.4 (35.4, 65.1)	0.15	54.8 (39.9, 69.4)	47.9 (33.5, 62.5)	0.08	54.0 (36.6, 68.7)	50.3 (37.9, 66.6)	0.71
Delta EDVi^d^ (ml/m^2^)	3.8 (−6.1, 12.6)	5.0 (−2.6, 12.9)	0.25	5.4 (−4.9, 12.6)	4.2 (−2.9, 13.5)	0.94	4.3 (−4.7, 11.9)	4.6 (−3.1, 13.8)	0.56	4.8 (−2.1, 12.3)	3.0 (−3.8, 12.9)	0.78
Delta ESVi^e^ (ml/m^2^)	−2.0 (−7.5, 5.3)	−0.5 (−6.8, 7.2)	0.22	−0.2 (−7.2, 6.1)	−1.9 (−7.8, 6.4)	0.85	−0.7 (−7.5, 6.3)	−1.5 (−7.7, 6.4)	0.81	−0.9 (−7.3, 5.5)	−1.0 (−7.9, 7.2)	0.84

Data are presented as median (25^th^, 75^th^ percentiles) or numbers (%). CCN2 was measured before and immediately after the PCI-procedure, at Day 1 (median 18.3 hours after PCI) and at 4-month follow-up in 249 STEMI patients. ^a^CMR: cardiac magnetic resonance imaging, ^b^LV: left ventricle, ^c^MVO: microvascular obstruction, ^d^EDVi: Indexed end-diastolic volume of LV, ^e^ESVi: Indexed end-diastolic volume of LV. P-values obtained by Mann-Whitney U test for continuous variables, Chi-square test for categorical variables.

Change in CCN2 levels (delta values) from hospitalisation to 4-month follow-up was weakly correlated with LVEF at 4 months (r_s_ = 0.15, p = 0.03), ∆LVEDVi (r_s_ = −0.19, p = 0.007) and ∆LVESVi (r_s_ = −0.14, p = 0.05), but these associations were present only at one sampling point (Day 1 for LVEF and before PCI for ∆LVEDVi and ∆LVESVi, respectively). There were no associations between change in CCN2 levels and final infarct size or myocardial salvage.

### Associations between CCN2 and adverse clinical events

The median follow-up time in the BAMI population was 55 months, and the median time to first clinical event was 16 months (1.3 years) after the index infarction. A total of 200 patients experienced an event, 66 deaths, 61 reinfarctions, 6 strokes, 52 urgent unscheduled PCI procedures and 15 hospitalisations with heart failure. In addition, 16 patients died after first having experienced a non-fatal event. As previously reported^16^, patients who experienced an event were significantly older, had higher frequency of previous MI, diabetes mellitus, heart failure, cerebrovascular disease, and had higher levels of admission glucose, N-terminal pro-B-type natriuretic peptide (NT-proBNP) and creatinine, than patients without events.

**Figure 2:**
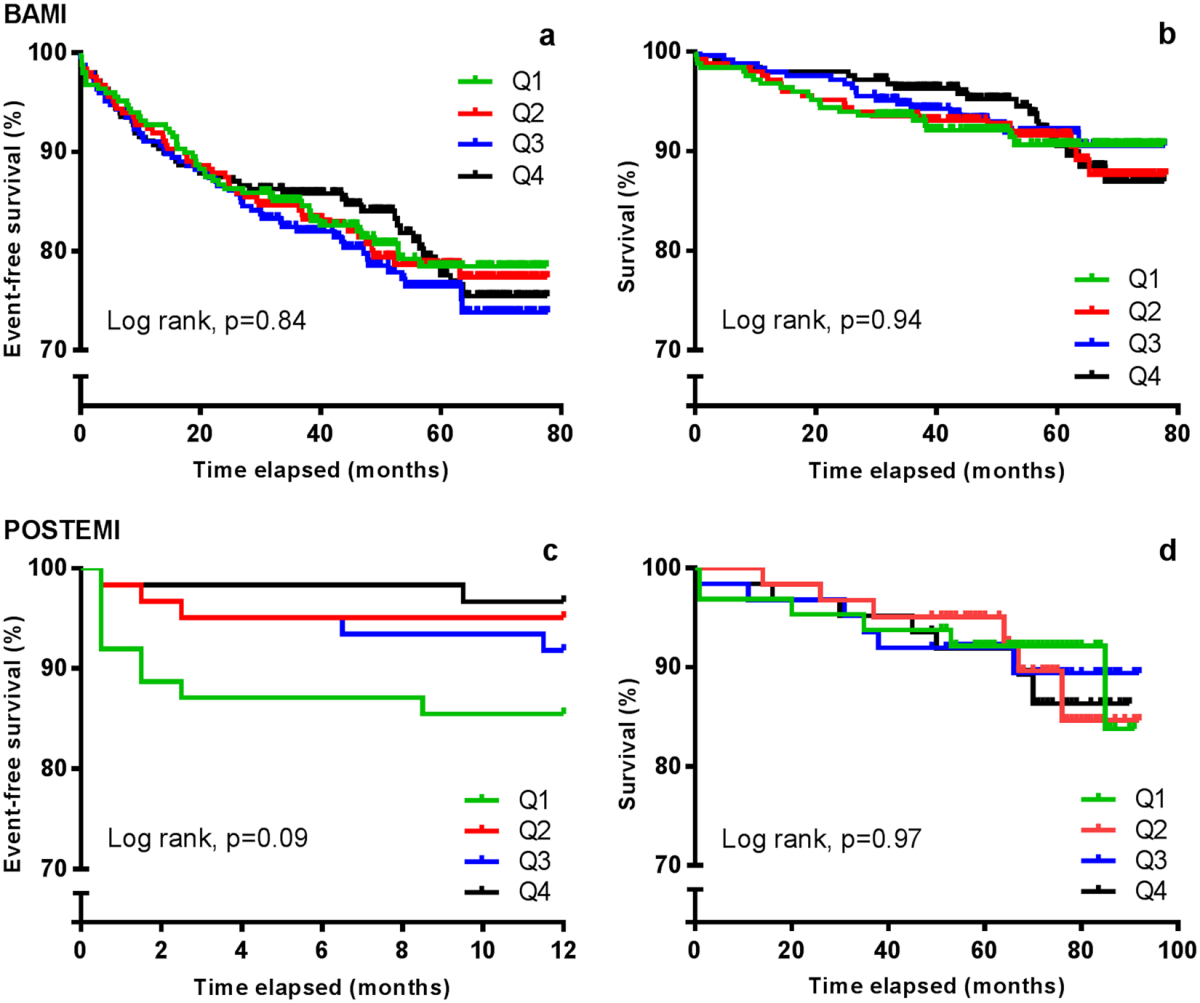
Adverse clinical events and overall survival according to quartiles of CCN2 measured median 18 hours after PCI in two STEMI cohorts. Upper panels (BAMI): CCN2 was measured in 988 patients in the BAMI cohort. A composite of clinical events (**a**) and all-cause mortality (**b**) were recorded with a median follow-up time of 55 months. Lower panels (POSTEMI): CCN2 was measured in 258 patients in the POSTEMI cohort. A composite of clinical events (**c**) was recorded after 12 months’ follow-up and all-cause mortality (**d**) was recorded after median 70 months’ follow-up.

There was no difference in CCN2 levels in patients who experienced a clinical event compared to patients without, nor was there any difference between patients who died compared to survivors (Table 3). Also, no significant trends across quartiles of CCN2 measured at Day 1 in risk of clinical events or total mortality were observed (Fig. 2). Furthermore, CCN2 levels were not associated with clinical events or all-cause mortality in Cox regression models, neither when analysed in quartiles nor dichotomised at median (Table 4).

**Table 3:** Levels of CCN2 according to adverse clinical events in two cohorts of patients with STEMI.

BAMI Study^a^	Clinical events (n = 200)	No clinical events (n = 788)	p-value	All-cause mortality (n = 66)	Alive at follow-up (n = 922)	p-value
CCN2 Day 1 (ng/ml)	27.6 (17.9, 51.3)	27.0 (17.6, 54.3)	0.85	26.3 (17.6, 51.2)	27.2 (17.7, 54.0)	0.61
**POSTEMI Study** ^**b**^	**Clinical events (n = 20)**	**No clinical events (n = 238)**	**p-value**	**All-cause mortality (n = 26)**	**Alive at follow-up (n = 232)**	**p-value**
CCN2 before PCI (ng/ml)	16.4 (12.5, 38.6)	27.0 (14.6, 61.0)	0.07	34.0 (15.8, 68.9)	26.0 (14.3, 53.9)	0.48
CCN2 after PCI (ng/ml)	18.0 (13.2, 29.5)	22.5 (14.9, 50.0)	0.16	28.8 (16.2, 59.3)	21.2 (14.2, 48.2)	0.37
CCN2 Day 1 (ng/ml)	14.7 (11.0, 34.5)	23.7 (13.2, 56.1)	0.06	27.9 (13.7, 60.0)	23.0 (12.5, 54.1)	0.49
CCN2 4 months (ng/ml)	—	—	—	27.7 (18.1, 70.1)	28.5 (18.1, 65.1)	0.83

Data are presented as median (25^th^, 75^th^ percentiles). A composite endpoint was defined as all-cause mortality, myocardial infarction, unscheduled revascularisation, hospitalisation with heart failure, or stroke. ^a^CCN2 was measured at Day 1 (median 18 hours after PCI) in the BAMI cohort. Follow-up time for both composite endpoints and all-cause mortality was median 55 months. ^b^CCN2 was measured before and immediately after PCI, at Day 1 (median 18.3 hours after PCI) and at 4-month follow-up in the POSTEMI cohort. Composite endpoints were registered after 12 months’ follow-up. All-cause mortality was registered after median 70 months’ follow-up. P-values obtained by Mann-Whitney U test.

**Table 4:** Associations between quartiles of circulating CCN2 measured at Day 1 and adverse clinical events in 988 STEMI patients (BAMI cohort).

	Composite endpoint (unadjusted) (n = 988) HR (95% CI)	p-value	Mortality (unadjusted) n = 988) (HR (95% CI)	p-value
**CCN2**				
Q2 vs Q1	1.04 (0.70, 1.55)	0.84	1.09 (0.60, 1.97)	0.80
Q3 vs Q1	1.17 (0.79, 1.72)	0.44	0.91 (0.49, 1.69)	0.76
Q4 vs Q1	1.00 (0.76, 1.50)	0.99	0.93 (0.51, 1.72)	0.83
Q3–4 vs Q1–2 (above or below median value)	1.06 (0.80, 1.40)	0.67	0.88 (0.57, 1.36)	0.58

CCN2 was measured median 18 hours after PCI. Composite endpoint was defined as all-cause mortality, myocardial infarction, unscheduled revascularisation ≥3 months after the index infarction, hospitalisation with heart failure, or stroke. Follow-up time for both composite endpoints and all-cause mortality was median 55 months. Hazard ratios (HR) and 95% confidence intervals (CI) obtained from Cox regression. Q1: lowest quartile, Q4: highest quartile.

Fewer adverse clinical events were registered in the smaller POSTEMI cohort. 20 patients (7% of total population) experienced an event during 12 months of follow-up (6 deaths, 3 reinfarctions, 2 urgent unscheduled PCI procedures and 9 hospitalisations with heart failure), and 26 deaths were registered (10% of total population) after median 70 months of follow-up. In line with the results from the BAMI cohort, CCN2 levels at all sampling points were similar in patients with and without clinical events (Table 3), and were not associated with adverse clinical events or all-cause mortality during long-term follow-up (Figure 2, Table 5).

**Table 5:** Associations between quartiles of circulating CCN2 and adverse clinical events in 258 STEMI patients (POSTEMI cohort).

	Composite primary endpoint (unadjusted) HR (95% CI)	P-value	Mortality (unadjusted) HR (95% CI)	P-value
**CCN2 before PCI**				
Q2 vs Q1	0.72 (0.23, 2.26)	0.57	1.07 (0.31, 3.69)	0.92
Q3 vs Q1	0.55 (0.16, 1.87)	0.34	1.47 (0.47, 4.63)	0.51
Q4 vs Q1	0.42 (0.11, 1.64)	0.21	1.78 (0.58, 5.44)	0.31
Q3–4 vs Q1–2	0.57 (0.22, 1.44)	0.23	1.57 (0.70, 3.49)	0.27
**CCN2 after PCI**				
Q2 vs Q1	1.21 (0.41, 3.61)	0.73	2.14 (0.63, 7.35)	0.23
Q3 vs Q1	0.48 (0.12, 1.92)	0.30	1.96 (0.57, 6.72)	0.28
Q4 vs Q1	0.67 (0.19, 2.38)	0.54	2.34 (0.70, 7.78)	0.17
Q3–4 vs Q1–2	0.52 (0.21, 1.30)	0.16	1.42 (0.65, 3.09)	0.38
**CCN2 Day 1**				
Q2 vs Q1	0.32 (0.09, 1.19)	0.09	1.17 (0.38, 3.64)	0.79
Q3 vs Q1	0.54 (0.18, 1.61)	0.27	1.13 (0.36, 3.49)	0.84
Q4 vs Q1	0.22 (0.05, 1.00)	0.05	1.31 (0.44, 3.89)	0.63
Q3–4 vs Q1–2	0.58 (0.23, 1.47)	0.25	1.13 (0.51, 2.47)	0.77
**CCN2 4 months**				
Q2 vs Q1			1.13 (0.33, 3.93)	0.84
Q3 vs Q1			0.94 (0.25, 3.51)	0.93
Q4 vs Q1			1.17 (0.34, 4.03)	0.81
Q3–4 vs Q1–2			0.99 (0.40, 2.44)	0.98

CCN2 was measured before and immediately after PCI, at Day 1 (median 18.3 hours after PCI) and at 4-month follow-up. Composite endpoint was defined as all-cause mortality, myocardial infarction, unscheduled revascularisation ≥3 months after the index infarction, hospitalisation with heart failure or stroke. A total of 20 patients experienced a composite endpoint during 12 months’ follow-up, and 26 deaths were registered after median 70 months’ follow-up. 5 patients died before the 4-month follow-up visit. Hazard ratios (HR) and 95% confidence intervals (CI) obtained from Cox regression. Q1: lowest quartile, Q4: highest quartile.

## Discussion

The main findings in the present study were that circulating CCN2 levels measured in the acute stages of STEMI were not associated with myocardial injury, impaired function or long-term prognosis.

We did not find any associations between CCN2 levels and various indices of myocardial injury and function, neither in the acute phase nor when measured 4 months after the index infarct. Furthermore, no associations were found between CCN2 and later cardiovascular adverse events or all-cause mortality in two different cohorts of STEMI patients. In accordance with this, CCN2 levels were similar in patients who experienced an adverse clinical event including all-cause mortality, compared to patients without.

While there is extensive evidence of CCN2 being a key mediator in cardiac fibrosis^17^, the role of CCN2 in MI and post-infarction remodelling remains unclear. In fact, previous reports have suggested both a pathological and protective role of CCN2. After an acute infarction, CCN2, activated by various mediators including TGF-β1, plays a role in regulation of fibroblast phenotype and function in the injured area^18^. Increased expression of CCN2 in cardiac myocytes and fibroblasts following MI has been demonstrated in several experimental models^2,10^, indicating a role of CCN2 in post-infarction fibrosis. One experimental study showed increased CCN2 expression in viable myocardium as late as 180 days after MI suggesting a role in late remodelling following MI^4^. Recent reports from animal models of IR-injury have discussed a cardioprotective role of CCN2. Both transgenic mice with cardiac-restricted overexpression of CCN2 and Langendorff-perfused hearts treated with recombinant human CCN2 showed enhanced tolerance towards IR-injury^13,14^. Moreover, one experimental study demonstrated that transgenic mice with overexpressed CCN2 had smaller infarcts, less LV dilatation and hypertrophy, and lower mortality, suggesting protective, anti-remodelling characteristics of CCN2 in MI^11^. In contrast to these results, we did not find any associations between circulating levels of CCN2 measured both before and after reperfusion and surrogate markers of IR-injury or LV remodelling including myocardial salvage, MVO and change in LVEDV in patients with STEMI. Neither was there any association with the iPost procedure used in the POSTEMI study^19^.

In a small human study of 42 STEMI patients, no overall changes of CCN2 levels from 2 days to 2 months and 1 year were found. However, when analysing patients according to increase or decrease during this period, patients with increasing CCN2 to follow-up had lower LV end-systolic volume and higher LVEF, suggesting that CCN2 activity after MI may attenuate remodelling^11^. There was, however, no information about CCN2 levels during the first 2 days after admission^11^. In the present study, we found a significant overall increase in CCN2 levels from hospitalisation to 4-month follow-up and delta CCN2 (hospitalisation to 4 months) was weakly correlated with changes in LV volumes. However, these associations were weak, not consistent across all the sampling points, and importantly, we did not find any associations with final infarct size or myocardial salvage.

Despite a potential role of CCN2 in the pathogenesis of post-infarction remodelling, we did not find any associations with adverse clinical events or all-cause mortality in either of the two cohorts. The consistent lack of associations between CCN2 and adverse clinical events in both the BAMI cohort, which had a relatively large number of adverse events and long-term follow-up, and the POSTEMI cohort with multiple sampling points, indicate that circulating CCN2 measured in the acute stages of STEMI does not add prognostic information for development of myocardial injury, heart failure, and later cardiovascular events. There are, to our knowledge, no other reports from large STEMI cohorts on CCN2 and long-term prognosis. A recent report, however, demonstrated that high baseline plasma levels of CCN2 were associated with increased risk of mortality and cardiovascular events, in patients with manifest, but stable, vascular disease^20^, in contrast to the hypothesis that high levels of CCN2 should be cardioprotective. It must be emphasised that this was a completely different patient population compared to our acute MI populations. The present results are based on CCN2 measured in the circulation. The lack of associations with myocardial injury and function obviously does not exclude an important role of locally acting CCN2 in the myocardium, both at the cardiomyocyte and the fibroblast level, which has been shown in numerous experimental studies^10,13^.

### Limitations and strengths

There are very few studies on circulating CCN2 in STEMI patients, and mainly with small cohorts. The strengths of this study are the relatively large cohort and long-term follow-up, the serial measurements in the acute phase of STEMI, the use of repeated CMR to evaluate myocardial function and injury, and the testing of any association with survival post-MI in two separate cohorts of STEMI patients.

Only one blood sample at Day 1 in the BAMI cohort and the lack of sampling between Day 1 and 4 months in POSTEMI are major limitations of this study. We may have missed the peak value, and it is possible that the main effects of CCN2 occur during the first weeks/months rather than hours/days after the infarction. The majority of previous research on CCN2 in MI has been experimental and on the cellular level. Although it seems that CCN2 is not a good circulating biomarker, we cannot exclude that locally released CCN2 in the myocardial tissue plays an important role in fibrosis and in the remodelling process of the infarcted myocardium. The relatively small number of adverse events in the cohort with serial blood sampling (POSTEMI cohort) increases the probability of a type 2 error. However, the similar results in the BAMI cohort with a larger number of events strengthen the conclusions. The relatively small infarct size judged by troponin T levels, few in-hospital deaths and relatively low incidence of heart failure in the BAMI population may indicate that this was a low-risk STEMI population, which may have influenced the results.

## Conclusion

Circulating levels of CCN2 measured early after STEMI were not associated with myocardial IR-injury, infarct size, adverse remodelling, impaired function or new adverse clinical events. The role of circulating CCN2 as a potential prognostic biomarker for adverse remodelling, heart failure development and long-term prognosis in STEMI patients appears to be limited.

## Methods

All parts of this study were approved by the Regional Committee for Medical Research Ethics, South-East Norway, and conducted in accordance with the ethical principles of the Declaration of Helsinki. All participants were included after providing written informed consent. The results from the POSTEMI and BAMI studies are reported in accordance with the CONSORT (CONsolidated Standards of Reporting Trials) 2010^21^ and STROBE (STrengthening the Reporting of OBservational studies in Epidemiology)^22^ checklists, respectively.

### Study populations

Two different cohorts of STEMI patients were studied in the present study. The Postconditioning in ST-Elevation Myocardial Infarction (POSTEMI) trial was a prospective, randomised, single-centre, open-label clinical trial investigating the effect of ischaemic postconditioning (iPost) on infarct size in STEMI patients. The trial was approved by the Regional Committee for Medical Research Ethics, South-East Norway, on July 30, 2008, and following a pilot period including 20 patients and evaluation of safety by the Data and Safety Monitoring Board the trial was registered at clinicaltrials.gov on June 16, 2009 (registration number NCT00922675). The study design including detailed CMR protocol has previously been reported in detail^19,23^, and the study flow-chart is available as Supplementary Figure S2. Briefly, 272 patients with first-time STEMI and symptom duration < 6 hours were included between January 2009, and August 2012, at Oslo University Hospital Ullevål, Norway. Patients with inability to provide informed consent, previous MI, renal failure (serum creatinine > 200 μmol/L), contraindications to CMR, and clinically unstable patients (cardiac arrest, cardiogenic shock, pulmonary congestion, or hypotension), were excluded. Two reperfusion strategies, primary PCI with iPost or conventional PCI, were compared. STEMI was defined and treated according to current guidelines^24^. CMR was performed median 2 days after the index event in the acute phase and repeated after 4 months’ follow-up, allowing assessment of infarct size, LVEF, LV volumes, myocardial salvage, and MVO. Estimation of myocardial salvage (%) was based on CMR measurements of myocardium at risk in the acute stage and final infarct size at 4 months’ follow-up, as previously reported^19^. Clinical end points were recorded at follow-up visits 4 and 12 months after the index infarction, and long-term mortality data were acquired from clinical records median 70 months after inclusion. The effect of iPost on the primary endpoint of the study, final infarct size measured by CMR after 4 months, was neutral^19^.

The other cohort included patients from the Biobanking in Acute Myocardial Infarction (BAMI) study which was an observational cohort study of stable patients with acute STEMI admitted to Oslo University Hospital Ullevål^25^. A total of 2760 patients with STEMI were admitted to the hospital in the inclusion period between July, 2007 and July, 2011, and 1025 patients were included in the BAMI cohort after written informed consent. The exclusion criteria were age below 18 years and patients who were otherwise unable (sedated) to sign written informed consent. STEMI was defined and treated according to current guidelines^24^. For recording of adverse clinical events, 988 patients were followed from inclusion until December 31, 2013, with a median follow-time 4.6 years. The clinical endpoints were registered by telephone contact or mail between June 1 and December 31, 2013. The remaining 37 patients were excluded as a result of not responding on contact (32) or a final diagnosis other than STEMI (5). For each endpoint, the hospital records were collected and then further evaluated by an Endpoint Committee. Deaths were obtained from the Cause of Death Registry, administered by the Norwegian Institute of Public Health. Questionnaires and hospital records at admission were used for basic clinical information.

### Definition of adverse clinical events

In both cohorts, a composite clinical endpoint was defined as death, MI, unscheduled revascularisation > 3 months after the index infarction, rehospitalisation with heart failure or stroke. PCI or coronary artery bypass grafting as a consequence of the index infarction, or repeated coronary angiography without a PCI procedure, were not considered as clinical events.

### Biochemical analyses

In the POSTEMI population, blood sampling was performed before (median 2.8 hours after symptom onset) and immediately after the primary PCI procedure and further at Day 1 at approximately 8 a.m. (median 18.3 hours after PCI), and at 4-month follow-up. In the BAMI population, blood samples were collected at Day 1 the following morning after an overnight fast, median 18 hours after PCI and 24 hours after symptom onset. Serum was prepared by centrifugation for 10 min at 2000 × g and samples were stored at −80 °C until analysed. Circulating levels of CCN2 were analysed by ELISA (R&D Systems Europe, Abingdon, Oxon, UK). Inter assay coefficient of variation (CV) was 10%, and samples from both cohorts were analysed simultaneously with the same lot number. Routine biochemical analyses, including C-reactive protein (CRP), NT-proBNP, and cardiac-specific troponin T were determined by conventional laboratory assays. Peak CRP and peak troponin T were defined as the maximum value measured during hospitalisation.

### Statistical analyses

Continuous variables are presented as median values (25, 75 percentiles) and categorical variables as proportions. Associations between CCN2 and clinical variables were assessed by both correlation analyses (Spearman’s rho) and group analyses (dichotomised or split into quartiles). Non-parametric tests were used throughout due to marked skewness in the distribution of CCN2. Mann-Whitney U test and Kruskal-Wallis test were used for group comparisons of continuous variables, while categorical variables were analysed using Chi-square test. Friedman test followed by Wilcoxon signed rank test were used to compare CCN2 levels at different sampling points. To investigate possible associations between CCN2 and adverse clinical events, the variable was split into quartiles (Qs) because linear effect could not be assumed. First, Qs 2, 3, and 4 were compared to Q1 in univariate Cox regression models. Next, patients with CCN2 above or below median were compared. A two-sided p-value < 0.05 was considered to be statistically significant. Due to the explorative nature of the study, no correction for multiple comparisons was performed. All analyses were performed by IBM SPSS Software, version 23.0 for Windows (SPSS Inc., Chicago, IL).

### Data availability

The datasets generated during the current study are available from the corresponding author on reasonable request.

## Electronic supplementary material

Supplementary Information
