# Supplementary material for: Evaluation of circulating levels of CCN2/connective tissue growth factor in patients with ST-elevation myocardial infarction
Source: Sci Rep. 2017 Sep 20;7:11945. doi: 10.1038/s41598-017-12372-w (PMC5607271; doi:10.1038/s41598-017-12372-w)
Supplement: Supplementary file 1 — Supplementary Information [file 41598_2017_12372_MOESM1_ESM.pdf]

Supplementary Information

Evaluation of circulating levels of CCN2/connective tissue growth factor in patients with ST-elevation myocardial infarction

Vibeke Ritschel[1,2,3,4,*,+], Christian Shetelig[1,2,3,4,+], Ingebjørg Seljeflot[1,2,3,4], Shanmuganathan Limalanathan[1,3,4,5], Pavel Hoffmann[1,6], Sigrun Halvorsen[1,3,4], Harald Arnesen[2,3,4], Jan Eritsland[1,3], Geir Øystein Andersen[1,2,3]

[1]Department of Cardiology, Oslo University Hospital Ullevål, Oslo, Norway

[2]Center for Clinical Heart Research, Oslo University Hospital Ullevål, Oslo, Norway

[3]Center for Heart Failure Research, Oslo, Norway

[4]Faculty of Medicine, University of Oslo, Oslo, Norway

[5] LHL Clinics, Feiring Heart Clinic, Feiring, Norway

[6]Section of Interventional Cardiology, Oslo University Hospital Ullevål, Oslo, Norway

[*]UXVINO@ous-hf.no

[+]These authors contributed equally to this work

Table of contents

Supplementary Table S1 3

Supplementary Figure S2 – POSTEMI Study flow diagram.. 4

Supplementary Table S1. Correlations between circulating CCN2 and myocardial injury and function assessed by CMR in the acute phase and after 4 months in 249 STEMI patients (POSTEMI cohort).

	CCN2 before PCI	p	CCN2 after PCI	p	CCN2 Day 1	p	CCN2 4 months	p
**CMR[a] in acute phase**								
Infarct size (% of LV[b]) mass)	0.05	0.45	-0.01	0.85	0.05	0.51		
Ejection fraction (%)	-0.03	0.66	0.01	0.83	-0.03	0.64		
Area at risk (% of LV)	-0.03	0.69	-0.10	0.16	-0.08	0.29		
Presence of MVO[c]	0.05	0.43	0.01	0.94	0.07	0.29		
**CMR after 4 months**								
Infarct size (% of LV mass)	0.03	0.65	-0.02	0.71	0.03	0.71	0.01	0.84
Ejection fraction (%)	-0.05	0.41	-0.01	0.85	-0.07	0.30	-0.03	0.69
Myocardial salvage (%)	-0.15	0.04	-0.10	-0.16	-0.14	0.06	-0.10	0.15
Delta EDVi[d] (ml)	0.08	0.27	0.04	0.59	0.07	0.30	0.01	0.88
Delta ESVi[e] (ml)	0.08	0.23	0.05	0.51	0.08	0.28	0.04	0.51

Data are presented as Spearman's rank correlation coefficient with p-value. CCN2 was measured before and immediately after the PCI-procedure, at Day 1 (median 18.3 hours after PCI) and at 4-month follow-up in 249 STEMI patients. [a]CMR: cardiac magnetic resonance imaging, [b]LV: left ventricle, [c]MVO: microvascular obstruction, [d]EDVi: Indexed end-diastolic volume of LV, [e]ESVi: Indexed end-diastolic volume of LV.

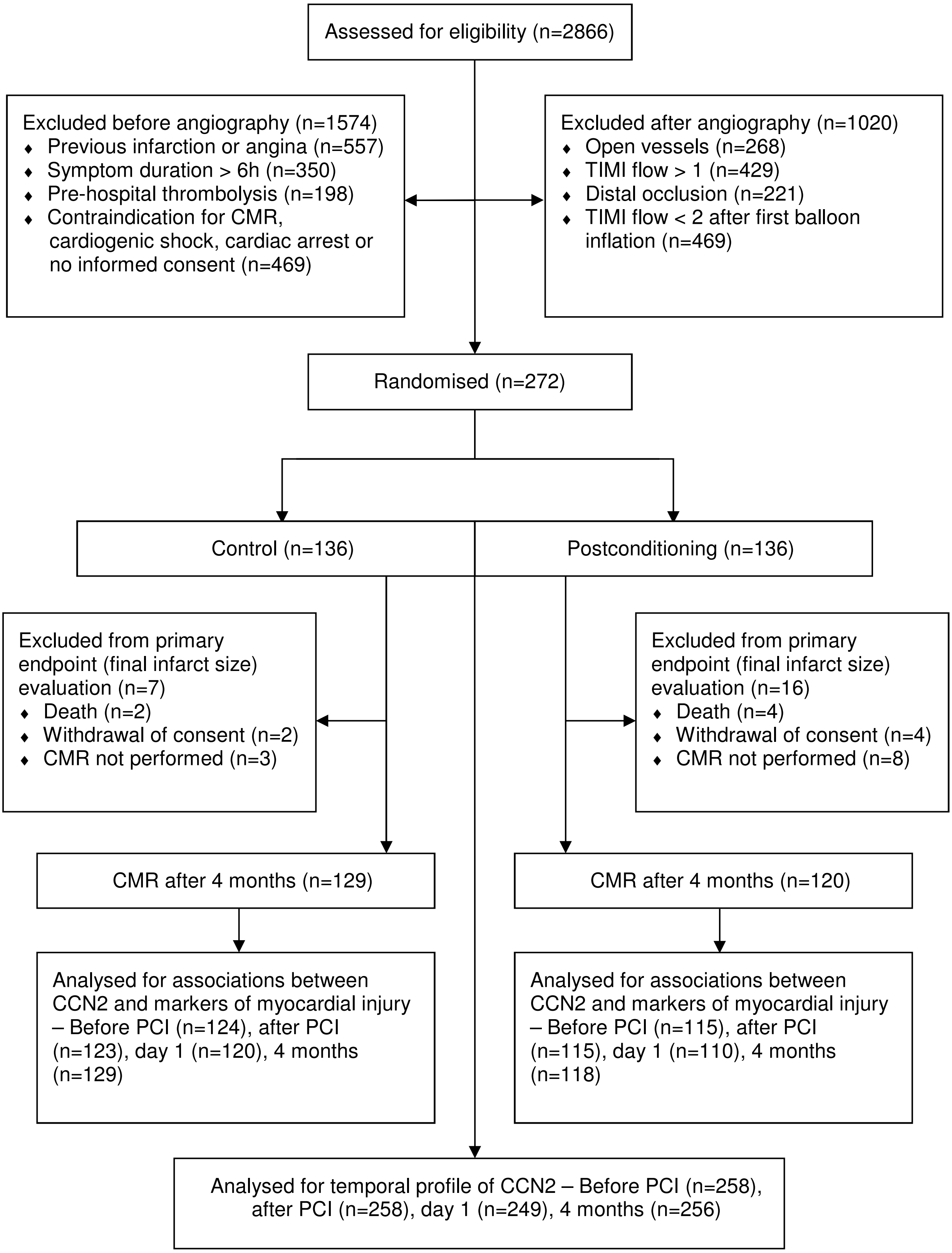

Supplementary Figure S2. POSTEMI study flow diagram.
